# Disturbances of Sperm Maturation and Minipuberty: Is There a Connection?

**DOI:** 10.1155/2014/912746

**Published:** 2014-03-13

**Authors:** D. Živković, I. Fratrić

**Affiliations:** ^1^Institute for Child and Youth Health Care of Vojvodina, 21000 Novi Sad, Serbia; ^2^Medical Faculty, University of Novi Sad, 21000 Novi Sad, Serbia

## Abstract

Male reproductive function in the general population raises an increased attention due to reports indicating declining sperm counts, increased occurrence of testicular cancer, cryptorchidism, and hypospadias. It is also hypothesized that prolonged exposure of the developing male, during both fetal and postnatal life, to exogenous estrogens could reduce Sertoli cell number and thus reduce sperm output (and sperm counts) in adult life. Fact is that infertility, which is defined as the inability to conceive after 1 year of unprotected intercourse, has a global prevalence of 9%. A male contributory factor is involved in approximately half of these cases, but most of the causes of reduced semen quality and other disturbances of male reproductive function are unknown. In the most affected men (azoospermic men) 15–20% had a prior history of cryptorchidism. The association between the cryptorchidism and infertility is one of the most studied potential causes of infertility. There are numerous studies that accentuate the importance of minipuberty for future fertility. Is it possible that a normal minipuberty ensures normal fertility despite malpositioned testes? And to move away from cryptorchidism, could impaired minipuberty be responsible for fertility problems in men who were born with both testes in their scrotal sacs?

## 1. Spermatogenesis

Spermatogenesis is a highly specialized process of cellular differentiation in which diploid progenitor cells of the testis differentiate into haploid spermatozoa. It begins with a differentiating cell division of diploid spermatogonial stem cells and continues with sequential cell divisions of spermatogonia and meiosis of spermatocytes to form round spermatids [1]. Successful differentiation of round spermatids into the complex structure of the spermatozoon is called spermiogenesis.

In humans, two morphologically distinguishable types of differentiated spermatogonia exist, called Ad (dark) and Ap (pale). Ad spermatogonia exhibit characteristics consistent with their identification as testicular stem cells, while the Ap spermatogonia exhibit characteristics of a progenitor cell [2].

Spermatogenesis occurs in the seminiferous tubules of the testis and relies on the appropriate expansion of undifferentiated and differentiated spermatogonia prior to the entry of germ cells into meiosis and subsequent spermiogenesis [3]. The process of spermatogenesis is under the general control of the endocrine system, and locally by a variety of direct and indirect interactions and signals between the developing germ cells and the surrounding microenvironment via autocrine/paracrine factors [4].

## 2. Testicular Dysgenesis Syndrome

Male reproductive function in the general population raises an increased attention due to reports indicating declining sperm counts [5, 6], increased occurrence of testicular cancer, cryptorchidism, and hypospadias [7, 8].

Some authors have hypothesized that these entities (declining semen quality, testicular cancer, cryptorchidism, and hypospadias) are symptoms of one underlying entity—the so-called testicular dysgenesis syndrome (TDS) [9]. They have suggested that an imbalance between estrogens and androgens during fetal life may be crucial. Initially, the estrogen hypothesis argued that increase of reproductive abnormalities in human males may be related to increased estrogen exposure in utero [10]. This hypothesis has been expanded to include endocrine disruptors, which include environmental toxins that can disrupt the hormonal balance of the fetus and thereby disturb sexual differentiation either by an estrogenic or an antiandrogenic effect [11].

## 3. Endocrine Disruptors

Reports on various experimental animals (e.g., sheep, rats, and mice) describe how exposure to exogenous estrogens during the neonatal period causes drastic reductions in the secretion of FSH from the pituitary gland and the presumption is that similar effects would occur before birth [12]. As FSH plays a vital role in controlling multiplication of Sertoli cells at this time [12] the prediction would be that estrogen-induced suppression of FSH levels would lead to a slower rate of Sertoli cell multiplication. As the number of Sertoli cells formed in fetal/neonatal life is an important factor influencing the maximum level of sperm production in adult life, the consequences of such a change in terms of sperm counts is obvious; moreover, such an effect is irreversible once Sertoli cell multiplication stops in early postnatal life. There is abundant evidence from man (hypogonadotropic hypogonadism) and from animal species that suppression of FSH levels in early postnatal life results in just such changes [13]. Recent evidence from the fetal sheep [14] also shows that suppression of FSH secretion in the fetal male during the second half of gestation results at birth in testes that contain 40% fewer Sertoli cells than occurs in control animals.

It is therefore hypothesized that prolonged exposure of the developing male, during both fetal and postnatal life, to exogenous estrogens (perhaps even at low levels) could reduce Sertoli cell number and thus reduce sperm output (and sperm counts) in adult life. Experiments involving exposure of rats to various xenoestrogens during the period of Sertoli cell multiplication have shown that in adult life such exposure results in small (8–12%) but highly significant reductions in testis size and a corresponding decrease in daily sperm production [15]. These effects have been achieved after exposure to relatively low levels of the chemicals (alkylphenols, phthalates; 1 mg/liter in drinking water of pregnant rats) under test. For example, butylbenzyl phthalate has been found to occur in butter and margarine at concentrations as high as 47.8 mg/kg [16]. Such findings suggest that there is the theoretical possibility that human exposure to such chemicals might have contributed to the decline in sperm counts in men described earlier.

After reading all the facts it is logical to conclude that the sperm quality is declining, and the disruptors are to blame. But is it really so? The Carlson meta-analysis, from which the declining sperm quality hypothesis started, was criticized subsequently for many methodological flaws. However, their controversial meta-analysis and hypothesis spurred public and scientific interest in the study of male reproductive function and stimulated more rigorous scientific work in the area. Subsequent research has not convincingly supported their hypothesis. There are few convincing data that men have become less capable of reproducing during the last century. In 1992, the world population was about 5.5 billion. In 2012, the world population exceeded 7 billion [17]. Most of this population increase occurred in poorer, densely populated countries where industrial effluent is poorly regulated or unregulated. For example, the Ganges delta region in India and Bangladesh has a rapidly growing population and is one of the most densely populated areas of the world. The Ganges River and its tributaries contain some of the most polluted water in the world. These pollutants include a variety of industrial solvents and by-products including biphenyls and other compounds postulated to be estrogenic endocrine disruptors [18, 19]. The substantial increase in world population, particularly in regions exposed to high concentrations of potential estrogenic pollutants, argues strongly against a deleterious effect of these pollutants on male fertility.

In humans, it is clear that in utero exposure to certain endocrine disruptors has significant adverse effects. The most famous example of a human endocrine disruptor is diethylstilbestrol, a synthetic estrogen that was used to treat nausea during the first trimester of pregnancy. Diethylstilbestrol has been associated with increased reproductive tract abnormalities in boys and vaginal adenocarcinoma in girls exposed to the drug in utero [20]. However, diethylstilbestrol was a drug administered to pregnant women. The data linking specific environmental pollutants and contaminants to reproductive tract abnormalities are tenuous. In the 2009 Endocrine Society Scientific Statement on Endocrine Disruptors, the authors list several compounds with estrogenic activity that might cause male reproductive tract disorders (including cryptorchidism, testicular cancer, and decreased spermatogenesis), but they acknowledge that the epidemiological data relating male reproductive tract disorders to environmental disruptors are indirect and that there is no direct evidence of endocrine disruptors' involvement in the pathogenesis of male reproductive tract disorders in men [19].

## 4. Minipuberty

Fact is that infertility, which is defined as the inability to conceive after 1 year of unprotected intercourse, has a global prevalence of 9% [21]. A male contributory factor is involved in approximately half of these cases [22], but most of the causes of reduced semen quality and other disturbances of male reproductive function are unknown [23]. In the most affected men (azoospermic men) 15–20% had a prior history of cryptorchidism [24]. The association between the cryptorchidism and infertility is one of the most studied potential causes of infertility. Even though it has been studied by many researches for many years many things are still quite unclear. It has been shown that two-thirds of patients with bilateral cryptorchidism and a third of patients with unilateral cryptorchidism [25] have problems with fertility. Why some of the patients with cryptorchidism are subfertile and others are not is still a mystery. Some answers have been found. For example it has been shown that “minipuberty” is a window of time in life of utmost importance for future fertility. The hypothalamic—pituitary—gonadal axis is transiently activated during the first months of postnatal life. In the healthy male infant gonadotropins, sex steroids and inhibin B increase to pubertal or even adult levels at 3 months of age, followed by a relatively quiescent period until reactivation of the hypothalamic—pituitary—gonadal axis in puberty [26–28]. The elevated inhibin B persists for a longer period of time than the elevated follicle-stimulating hormone (FSH), luteinizing hormone (LH), and testosterone [29]. The initial activation of the HPG axis is believed to be important for genital development, including renewal and differentiation of the germ cells. Patients with cryptorchidism have smaller increase in testosterone level compared to healthy boys [26]. Problems with fertility are not related to the testicular position, since some patients have fertility problems in adulthood even after a successful surgery at an early age [30]. Disturbed “minipuberty” could be responsible for future fertility problems, as it has been shown that cryptorchid patients with similar testicular position, timing of surgery, and testicular histology have different sperm counts depending on whether they received postoperative hormonal support or not [31]. There are clearly two subgroups of cryptorchid boys. Those with a sufficient Leydig cell secretory capacity have near normal testicular histology (present Ad spermatogonia) at the time of surgery, and they are not expected to have problems with fertility [32]. Those with a suboptimal Leydig cell secretory capacity have low Ad spermatogonia count at the time of surgery, and consequently poor prognosis for future fertility, despite successful surgery [33]. For now there is no precise way to noninvasively determine to which group a patient belongs. Testicular biopsy is the only diagnostic procedure capable of identifying patients who need to be treated with LH-RH analogue following successful orchidopexy and should therefore be routinely performed during the surgery. Ad spermatogonia proved to be a discriminating factor with respect to the fertility outcome [34]. The presence of Ad spermatogonia in the undescended testis influences the levels of FSH, and in patients with unilateral cryptorchidism the presence of Ad spermatogonia in the contralateral descended testis is a dominant predictor for the fertility outcome, since in boys with unilateral cryptorchidism, testicular pathology caused by hormonal imbalance is bilateral; 71% of scrotal testes have a reduced number of germ cells and 75% have impaired gonocytes transformation into Ad spermatogonia [34].

## 5. Conclusion

All of the above findings accentuate the importance of minipuberty for future fertility. Is it possible that a normal minipuberty ensures normal fertility despite malpositioned testes? And to move away from cryptorchidism, could impaired minipuberty be responsible for fertility problems in men who were born with both testes in their scrotal sacs? Many different opinions have been addressed in this review. The aim of it is not to show what is right, but to awake a curiosity in as many researchers dealing with male fertility, sex hormones, and cryptorchidism to continue to study these problems with a mind as wide open as possible.
